# Cancer prevention, screening, and survivorship ECHO: A pilot experience with an educational telehealth program

**DOI:** 10.1002/cam4.4421

**Published:** 2021-11-24

**Authors:** Tyler S. Severance, Zheng Milgrom, Anyé Carson, Caitlin M. Scanlon, Rishika Chauhan O’Brien, Brent Anderson, Mary Robertson, Andrea Janota, Scott L. Coven, Eneida A. Mendonca, Joan Duwve, Terry A. Vik

**Affiliations:** ^1^ Riley Hospital for Children Indianapolis Indiana USA; ^2^ Indiana University School of Medicine Indianapolis Indiana USA; ^3^ Riley Hospital Division of Pediatric Hematology Oncology Indianapolis Indiana USA; ^4^ Indiana University Richard M. Fairbanks School of Public Health Indianapolis Indiana USA; ^5^ Regenstrief Institute Indianapolis Indiana USA; ^6^ Indiana Cancer Consortium Indianapolis Indiana USA; ^7^ Indiana State Department of Health Indianapolis Indiana USA

**Keywords:** cancer, disparities, education, public health, telehealth

## Abstract

**Introduction:**

The American Cancer Society, Inc. (ACS) estimates that 37,940 Indiana residents were diagnosed with cancer in 2020, which remains the leading cause of death in the state. Across the cancer continuum, national goals have been established targeting recommended benchmarks for states in prevention, screening, treatment, and survivorship. Indiana consistently falls below most goals for each of these targeted categories.

**Methods:**

To address these disparities, we implemented Project ECHO (Extension for Community Healthcare Outcomes) as a virtual telehealth educational platform targeted at local healthcare providers. ECHO programs utilize a novel tele‐mentoring approach to the education of clinicians in a hub/spoke design. Sessions occurred twice monthly from September 2019 to September 2020 and consisted of a traditional didactic lecture and a case‐based discussion led by participating providers.

**Results:**

During the pilot year there were a total of 22 ECHO sessions with 140 different participants. On average, 15.5 spokes attended each session with increasing participation at the end of the year. Post‐session surveys suggested generally favorable perception with 72% of respondents finding the quality “excellent.”

**Discussion:**

Given the increasing rate of recurrent participation toward the end of the pilot year in conjunction with the favorable survey responses following each session, it was felt that the program was overall successful and warranted continued implementation.

**Conclusion:**

The Project ECHO platform is a validated telehealth education platform that has the potential to impact cancer care at multiple points along the cancer continuum at the regional level.

## Graphical abstract

Project ECHO is a telehealth education platform seeking to improve the care of chronic diseases in medically underserved communities. Here, we describe the implementation of this platform to address cancer prevention, screening, and survivorship.

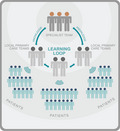

## 1 BACKGROUND AND INTRODUCTION

The American Cancer Society, Inc. (ACS) estimates that 37,940 Indiana residents were diagnosed with cancer in 2020, amounting to more than three new cases of cancer diagnosed every hour of every day.^1^ Although cancer remains the second leading cause of death in Indiana, the rate of cancer survivorship has continued to improve.^2^ The Indiana Cancer Consortium (ICC) was founded to “reduce Indiana's cancer burden through the coordinated, collective action of its members and the sharing of resources, knowledge, and passion.”^3^ With funding and support, in part, from the Indiana Department of Health (IDOH), and with healthcare partnerships across the state, the organization now includes more than 400 members and has grown to reach medically underserved areas to help improve access to high‐quality cancer‐related care.^3^

Across the cancer continuum, national goals have been established targeting recommended benchmarks for states in prevention, screening, and survivorship.^3^ Indiana consistently falls below most goals for each of these targeted categories. Prevention via obesity mitigation, vaccination, and smoking cessation are all below national goal thresholds. Rates of appropriate cancer screening for breast, cervical, colorectal, and lung cancers fall at least 10%–15% short of target levels in Indiana. Finally, statewide survivorship markers remain below goal thresholds in regards to poor mental health, poor physical health, achieving healthy weight, mitigating tobacco use, and various other categories.^4^ It will take broad training and outreach to affect statewide change; however, an educational program, focused on addressing these disparities at the level of the local primary care provider, can help achieve these goals.

**FIGURE 1:**
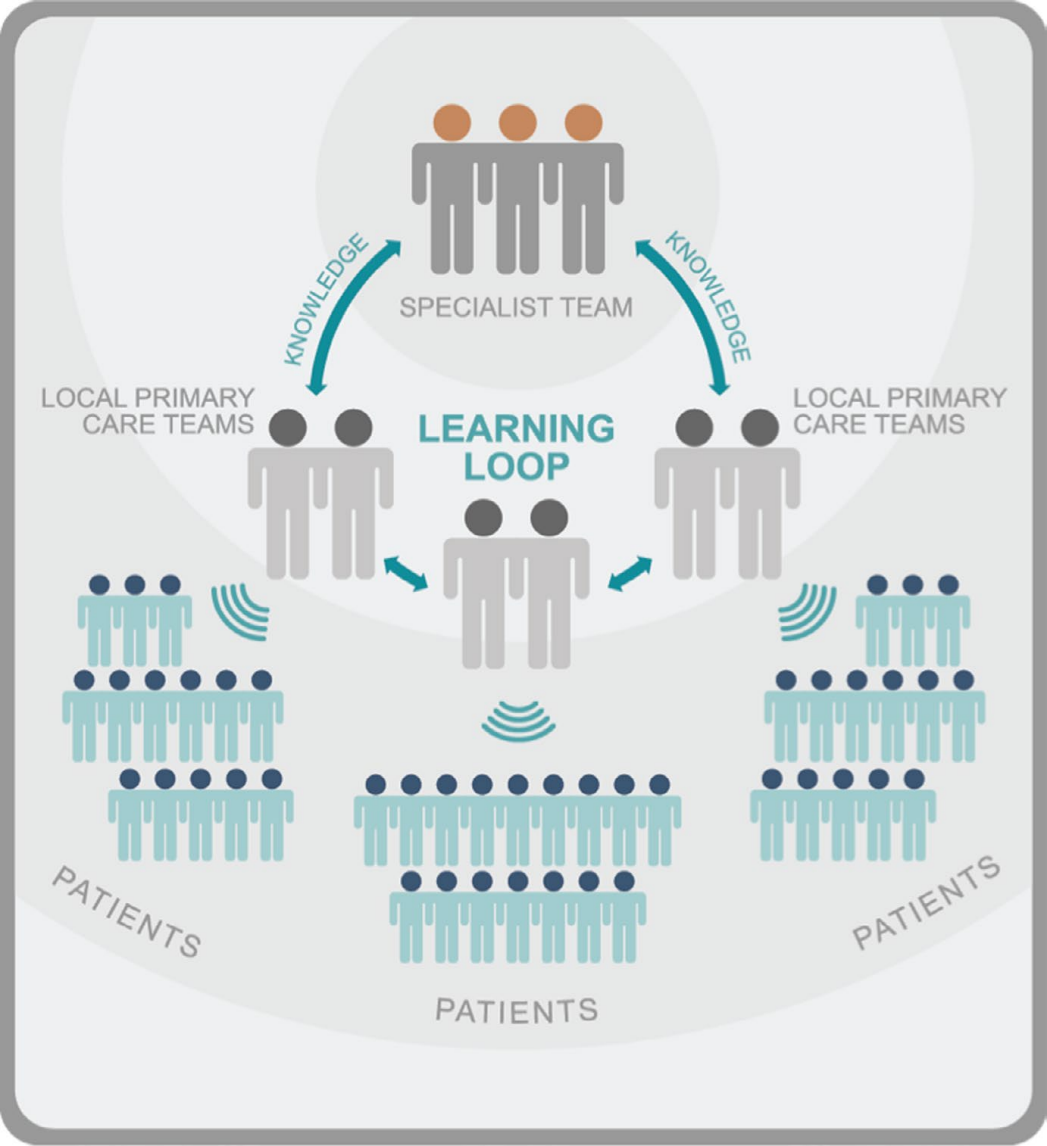
A representation of the educational learning loop where a bidirectional flow of knowledge can impact an exponentially greater number of patients. Reproduced with permission from the ECHO Institute^7^, ^8^

To address these disparities at the local provider level, the Indiana University Richard M. Fairbanks School of Public Health ECHO Center, the ICC, and IDOH partnered to implement a Cancer Prevention, Screening, and Survivorship Care curriculum to be delivered using the Project ECHO framework (ECHO; Extension for Community Healthcare Outcomes).^5^, ^6^, ^7^ ECHO programs utilize a novel tele‐mentoring approach to the education of clinicians in a hub/spoke design. The goal of ECHO is to train providers, not treat patients; the providers are the spokes and no patient is ever on the network. TeleECHO clinics typically convene weekly, bi‐weekly, or monthly for 60–90 min sessions during which deidentified case presentations, demonstrations, and didactics are provided synchronously and recorded for asynchronous observation. All knowledge is shared in a learning loop at no cost to participants and is represented in Figure 1.^7^, ^8^ All participants, including both the spoke site providers and the hub team specialists, are actively engaged and learning from the experience. Continuing medical education credits are provided based on clinical specialty. The ECHO Institute was founded in Albuquerque, New Mexico, and is supported by generous grants and funding from the state of New Mexico and various philanthropic organizations.^7^ The ECHO Institute offers training to teams from around the world desiring to replicate the ECHO model and their results are validated for various disease types.^8^, ^9^, ^10^, ^11^, ^12^ The goal of each ECHO varies based on the needs of the spoke sites and the disease type. For cancer‐related care in Indiana, the ECHO focused on prevention, screening, and management of survivorship care.

## 2 METHODS

Prior to launching the Indiana Cancer ECHO, planning and infrastructure were required to build an effective hub team. Several subject‐matter experts (SMEs) partnered with previously established ECHO program leadership at our institution. The SMEs attend a specialized immersion training in Albuquerque, New Mexico, to learn about the principles and mechanics of building and implementing a new ECHO program. The hub team was established to help cover each of the primary cancer‐related goals that were identified during the initial investigation. This team included a multidisciplinary group of specialists, including those with backgrounds in pediatric and adult oncology, survivorship care, palliative care, adult and child psychiatry, cancer epidemiology, public health, family medicine, nutrition, and social work. Also included in our hub team was a cancer survivor, a nurse practitioner with significant experience in cancer care, a clinical oncology nurse, an ECHO coordinator, and members of the state department of health. A curriculum was designed to best target recognized disparities in the region and was spread over 24 planned sessions to encompass the calendar year of the pilot experience.

Once the hub team was built, recruitment of spoke sites was achieved through various mechanisms. The hub team and collaborators, such as the ICC, utilized email invitations as the primary recruitment pathway. Professional societies such as the ACS, the American Academy of Family Physicians, and the American Academy of Pediatrics were incorporated into our community outreach in addition to the ICC network consisting of prior meeting attendees and Indiana health stakeholders. Anyone who functions as part of the healthcare team was included in invitations to participate in teleECHO clinics. Potential participants comprised of anyone broadly defined as a healthcare worker including physicians, physician assistants, nurse practioners, registered nurses, psychiatrists, psychologists, social workers, community health workers, pharmacists, patient navigators, care coordinators, emergency medical technicians, pastoral care, healthcare educators, clinic administrators, and medical legal partners. An employee with marketing experience was contracted by the hub team, received training in New Mexico, and coordinated with various media groups during the initial launch phase to generate additional publicity.

The initial hub team location operated out of the School of Public Health. The hub team room was set‐up using two monitors, a microphone, speaker, camera, and a computer with high‐speed internet. TeleECHO sessions took place via real time, interactive videoconferencing, using a HIPAA‐compliant, cloud‐based software application called Zoom. Zoom was made available at no cost to participants. Spoke site participants could use a computer, mobile device, tablet, or phone to connect to the conferences. During the pilot year, the COVID‐19 pandemic necessitated adaptation by the hub team to comply with institutional limits on in‐person meetings. Therefore, hub members also connected remotely for the last 6 months of the year.

Each ECHO session was designed based on the structure proposed by the founding leaders in the ECHO community and contained a brief 20–25 min didactic session followed by a case discussion. The call for cases was announced 2–5 days prior to each session with electronic or phone options for submission. Prior to the session, the hub team members were given the completed and deidentified case template as well as the didactic slides for preview. Subspecialty experts were invited to attend as guests of the hub team as needed. During each session, the iECHO program, an automated parallel data collection software, was used to track participation information and demographics related to cases.

To continuously evaluate the quality of the sessions and spoke participant experiences, surveys were sent electronically prior to and immediately following each ECHO session. Completion was not required but strongly encouraged. These surveys were designed to evaluate the quality of the preceding session and offer qualitative feedback for future didactic topics. Surveys were sent within 24 h of each completed ECHO session and a verbal reminder was stated at the end of each session. In addition to the electronic surveys, participants were also given the opportunity to receive a range of continuing education (CE) credit or Certified Health Education Specialists (CHES) credit via text response after each session. CE was specifically awarded based on participation and survey response was not a requirement for CE. Several members of the hub team met at quarterly intervals to review recent trends in participation and incorporate feedback from surveys.

At the conclusion of the 1‐year pilot experience, the results of all surveys were compiled, as were the data on participation and case submissions. This was reviewed by the hub team to help plan the upcoming year's curriculum and to continuously adapt and improve the program.

## 3 RESULTS

**FIGURE 2:**
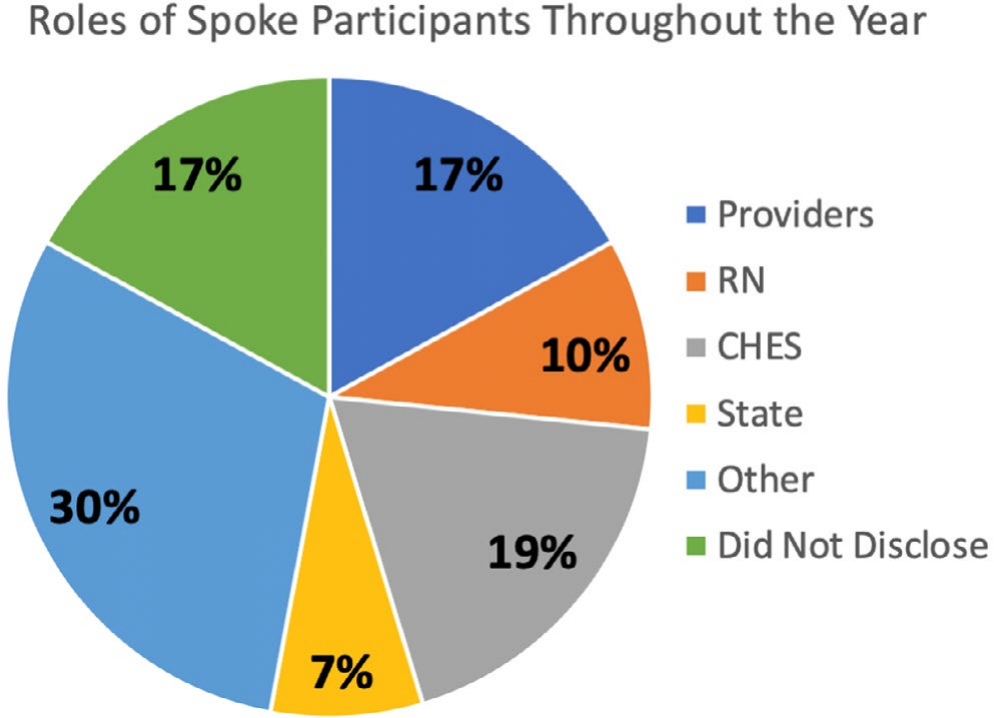
Graphic represenation of the total distribution of provider roles for participants at all ECHO sessions

**FIGURE 3:**
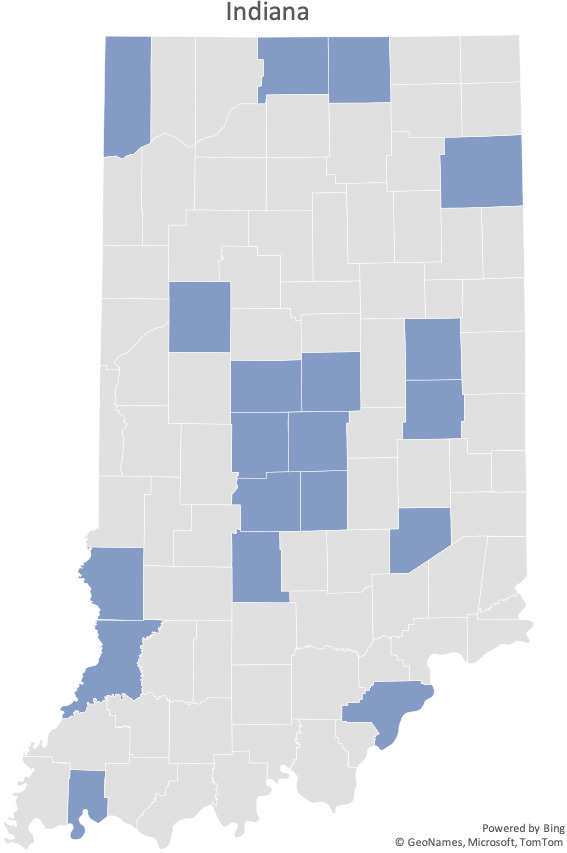
State map of Indiana where highlighted counties represent those with participation in Cancer ECHO sessions. Map generated using Microsoft Excel

In the months leading up to launch, the primary hub team members recruited several additional specialists to cover the various anticipated needs of the spoke sites (Complete hub team list is available in Table S1). A curriculum was constructed which attempted to address the most impactful disparities affecting the state of Indiana (Table S2).^4^ Subject‐matter experts were recruited to deliver these didactic sessions. Recruitment was performed and ultimately resulted in 270 enrolled participants with 140 participants attending at least one session over the course of the year. These included individuals from various roles and are shown in Figure 2. In scrutinizing the breakdown of medical providers, 69% were physicians with the remainder being advanced practice providers. The recruited participants included representation from 75 different zip codes both inside and outside the state of Indiana. A total of 64% of spoke attendees were from the state of Indiana while an additional 9% of participants were from a neighboring state. The ECHO program also drew international awareness with three different countries participating––Kenya, Nigeria, and Lebanon. A map of local participation by county is noted in Figure 3.

**FIGURE 4:**
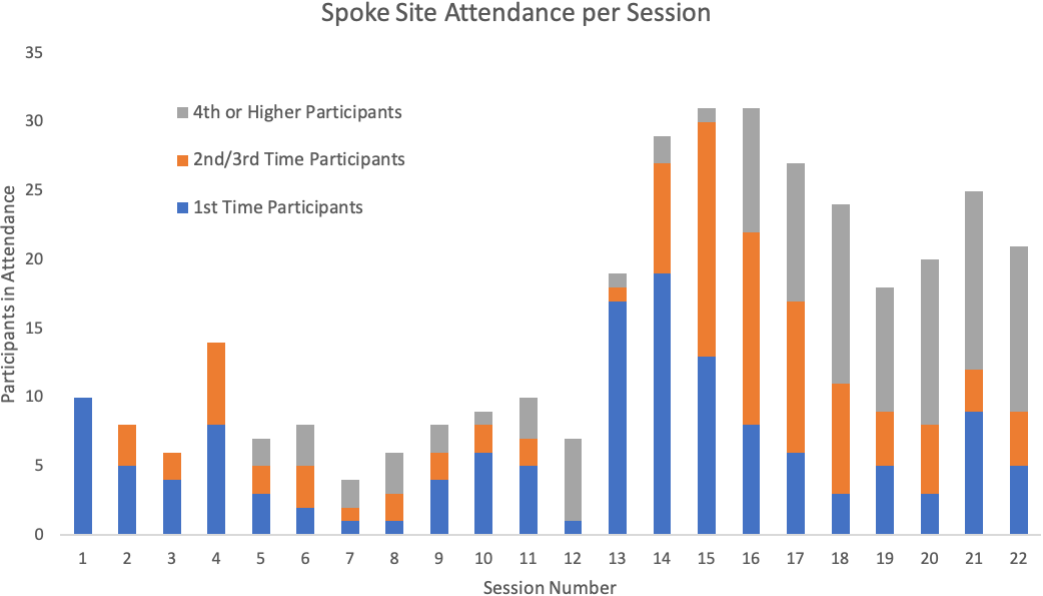
A graphic representation of spoke site attendance at each of the 22 sessions during the year with segments representing recurrent participation

The ECHO program consisted of 22 twice monthly sessions during the course of the year. One session was canceled in the spring of 2020 due to obligations from multiple hub and spoke team members to address the emerging COVID‐19 pandemic. An additional session was missed at the end of the pilot year so hub team members could plan for the next year of implementation. The ECHO sessions averaged 15.5 spoke attendees, with a gradual increase in participation during the latter half of the year. Longitudinal representation of spoke attendance frequency is provided in Figure 4. Regarding hub team participation, an average of 11 hub team members attended each session and are not included in Figure 4. All but one ECHO session had both a didactic and a case presentation. A scheduling miscommunication prevented one participant from being able to present their case.

Prior to each ECHO session, a brief survey was sent to participants assessing the impact of the prior ECHO clinic. We received 69 responses during the pilot year. Participants remarked that the most memorable sessions were the HPV vaccination presentation, the oncology fertility didactic, the session on patient‐centered language, and trauma‐centered dialog. Eighty‐three post‐session surveys were completed (24% response rate). Those surveyed viewed the session favorably with 64% saying the stated objectives were met “very well” and 72% indicating that the quality of the session was “excellent.” Among those surveyed, no participants reported the objectives were met "not well" or "slightly well" and no one felt the session was of “terrible” or “poor” quality.

## 4 DISCUSSION

The Cancer Screening, Prevention, and Survivorship ECHO was built to address multiple points of need across the cancer continuum. Each of these principles was recognized in the initial statewide needs assessment conducted by the ICC and the IDOH. This assessment allowed for better, more responsive curriculum design as well as more appropriate recruitment of specialists for the hub team and positioned the ECHO well for the successful delivery of education throughout the pilot year.

The ECHO model itself is based on the combination of didactics with case‐based learning. The time allocated for each session––approximately 20 min for the didactic and more than an hour for case discussion––reflects the critical importance of bidirectional and engaged learning. Throughout the year‐long experience, we followed this model very closely with rare deviations only due to uncontrollable situations (lecturer canceled or a case presenter had unforeseen clinic responsibilities). Initially, it was challenging to find suitable cases from the spoke sites. For sessions with no volunteered cases, the hub team members used a potentially beneficial case from their own clinical practice to help generate dialog.

One of the most powerful indicators of success for this program was the increasing rate of recurrent participation toward the end of the year (Figure 4). In our opinion, this demonstrated that the program was of sufficiently high academic value and participants were motivated to attend 60–90 min calls twice each month, and continue coming back to participate. This was a positive shift from the lower rates of attendance, both new participant and returning participant, during the initial months of the pilot. As time passed, the program became increasingly more effective, resulting in increased participation and improved retention.

Although the ECHO program was initially targeted toward primary care providers, it became quickly evident that many other members of the healthcare team were interested in the program. This diverse spoke participation lent itself to a truly multidisciplinary dialog and pushed the hub team to adapt case discussions and didactics to remain relevant for all participants. We feel this adaptation to accommodate all learners helped reinforce continued participation during the latter half of the ECHO.

As our team attempted to evaluate the ECHO after each session, it became clear that surveys were only partially effective as a feedback tool. We noted moderate engagement with surveys as only 22% of possible surveys were completed. Reminders were given to promote survey utilization at each ECHO session as well. Despite the low completion rate, the surveys provided the team with actionable feedback. There were topic suggestions for future didactic lectures as well as modifications to the ECHO flow––such as limiting the length of the didactics themselves––that were incorporated into the ECHO model as feedback became available. Adapting to the needs of our spoke participants in real time was a critical part of our growth. Without a mechanism for qualitative evaluations in real time, this improvement opportunity may have been missed. To enhance participant feedback, the ECHO team will include multiple assessment modalities moving forward. These may include spoke site participant focus groups or semi‐structured interviews.

The ECHO program also showed its flexibility in the midst of the global COVID‐19 pandemic.

At the halfway point––coinciding with session 13––the COVID‐19 pandemic was rapidly expanding locally, necessitating a canceled session to permit providers time to adapt to a changing medical community. The hub team transitioned to a virtual work environment and the ECHO resumed with increasing participation from spokes demonstrating that the virtual platform was adaptable and well‐suited for continued educational outreach during the pandemic. Furthermore, despite newly imposed financial restrictions, ECHO programs continued at our institution due to the low cost for continued use.

Building on lessons learned during the pilot year, the hub team intends to launch a second year of Cancer ECHO programming. There will be a dedicated interval to gather qualitative and quantitative feedback for the entire pilot project and additional time for planning sessions to incorporate this feedback into the next year‐long curriculum. The extra time will also allow for further recruitment across the state. In addition, it is recognized that although the ECHO platform is an educational program, the true value would be best recognized in the gradual improvement in public health in medically underserved communities. This represents a long‐term goal of the program and efforts will be made a continued point of emphasis in the IDOH, ICC, and ECHO planning forums.

Overall, the spoke site participants and the hub team members indicated that the program was a success. Increased participation with higher continuation rates at the end of the year and positive responses in the feedback surveys reflect a favorable experience. There were challenges that the ECHO team will need to address if they chose to continue the program, including better identification of appropriate cases for presentation and incorporating both short‐ and long‐term feedback. However, the virtual learning platform creates a robust and engaging forum for education and mentorship, and its use in the cancer community seems warranted. With appropriate partnerships from the IDOH and the ICC, the ECHO program was poised for successful implementation.

## 5 CONCLUSION

The Project ECHO platform is a validated telehealth education platform that has the potential to impact cancer care at multiple points along the cancer continuum at the regional level. After launching in 2019, the program operated for 1 year without significant deviations from the ECHO framework. Given the sustained and recurrent participation, coupled with the positive feedback from participant surveys, the ECHO launch and implementation was successful.

## CONFLICT OF INTEREST

None.

## AUTHORS CONTRIBUTION

All authors contributed to the conceptualization, design, and implementation of the research, to the analysis of the results, and to the writing of the manuscript. In addition, all authors participated in response to the reviewers and contributed to the revised manuscript.

## ETHICAL APPROVAL

This study was approved by the Indiana University Institutional Review Board.

## Supporting information

Table S1

Table S2

## Data Availability

The data that support the findings of this study are available from the corresponding author upon reasonable request.
