# Supplementary material for: Cancer prevention, screening, and survivorship ECHO: A pilot experience with an educational telehealth program
Source: Cancer Med. 2021 Nov 24;11(1):238–44. doi: 10.1002/cam4.4421 (PMC8704156; doi:10.1002/cam4.4421)
Supplement: Supplementary file 1 — Table S1 [file CAM4-11-238-s002.docx]

| **Session Number** | **Didactic Topics** | **Curriculum Focus** |
| --- | --- | --- |
| 1 | ECHO Orientation  Local Survivor Story | N/A |
| 2 | Talking with Parents about HPV | Prevention, Screening |
| 3 | Pediatric Palliative Care and Bereavement | Survivorship |
| 4 | Cancer Screening Recommendations | Screening |
| 5 | Motivational Interviewing | Prevention, Screening |
| 6 | Smoking Cessation | Prevention |
| 7 | Nutrition for Cancer Prevention | Prevention |
| 8 | Adolescent and Young Adult Survivorship Guidelines | Survivorship |
| 9 | Cervical Cancer Prevention and Screening | Prevention, Screening |
| 10 | Family Psychosocial Stresses of Cancer Survivorship | Survivorship |
| 11 | Goshen Cancer Center – a Community Model for Comprehensive Cancer Care | Prevention, Screening |
| 12 | Oncofertility in the Cancer Survivor | Survivorship |
| 13 | Canceled – COVID-19 response | N/A |
| 14 | Financial Toxicity for Survivorship | Survivorship |
| 15 | Men's HPV cancers | Prevention |
| 16 | Brief Action Planning | Prevention |
| 17 | Trauma Informed Care | Screening, Survivorship |
| 18 | Immunizations as Cancer Prevention | Prevention |
| 19 | "Breast Cancer as a model for Studying Health Disparities and Social Determinants of Health" | Prevention, Screening, Survivorship |
| 20 | Effective Use of Sensitive Language | Survivorship |
| 21 | Expanding the Scope of Survivorship in Rural Tribal Communities* | Survivorship |
| 22 | Colorectal Screening during COVID | Screening |
| 23 | Managing cancer risk in patients with a history of HCV | Screening |
| 24 | Planning Session | N/A |

Table 1: Didactic topics for each of the sessions of the ECHO curriculum as well as their emphasis on the cancer continuum

*Didactics led by Spoke Sites
