# Supplementary material for: Cancer prevention, screening, and survivorship ECHO: A pilot experience with an educational telehealth program
Source: Cancer Med. 2021 Nov 24;11(1):238–44. doi: 10.1002/cam4.4421 (PMC8704156; doi:10.1002/cam4.4421)
Supplement: Supplementary file 2 — Table S2 [file CAM4-11-238-s001.docx]

| Role | Credentials |
| --- | --- |
| Facilitator | Physician, Fellow |
| Peds Oncologist | Physician |
| Adult Oncologist | Physician |
| Cancer APP | APN |
| Survivorship Clinic Liaison | Physician |
| Note Taker | ICC Director |
| Clinical Psychologist | PhD, HSPP |
| Oncology Dietitian | RDN, CSO, CD |
| Pediatric Palliative Care Social Worker | MSW, LCSW |
| Community Liaison | ACS Member |
| ISDH Cancer Educator | Practice Coach |
| Cancer Disparities SME | Director Health Equity (CTSI) |
| ECHO Center Director | MPH |
| ECHO Program Coordinator | MPH |

Table 2: The composition of the hub team and provider roles at the time of ECHO launch
